# Pattern of cardiovascular admissions at Nnamdi Azikiwe University Teaching Hospital Nnewi, South East Nigeria

**DOI:** 10.11604/pamj.2014.17.116.1837

**Published:** 2014-02-18

**Authors:** Charles Ukachukwu Osuji, Emmanuel Ikechukwu Onwubuya, Gladys Ifesinachi Ahaneku, Emeka Godwin Omejua

**Affiliations:** 1 Faculty of Medicine, College of Health Sciences, Nnamdi Azikiwe University, Nnewi Anambra State, Nigeria

**Keywords:** Cardiovascular disease, cerebrovascular accident, heart failure, hypertension

## Abstract

**Introduction:**

Cardiovascular disease (CVD) is one of the top killer diseases in the world sparing neither developed or developing countries. The study was carried out to determine the pattern of cardiovascular admissions at Nnamdi Azikiwe University Teaching Hospital Nnewi South East Nigeria.

**Methods:**

The study was a retrospective study covering the period January 2007 to December 2009. SPSS version 13 software was used to analyze data.

**Results:**

537 (15%) patients were admitted into the study out of 3546 patients {females 1756 and 1790} admitted into medical wards. 322 (60%) of study population were males and 215 (40%) females. 359(67.5%) were discharged, 170 (32%) died and 8 (0.5%) were discharged against medical advice. The majority of the deaths 105(61.8%), were in patients with CVA. Most of the deaths (111 or 65.3%) occurred within the first seven days of admission. The mean age of the population was 60.7 years ±15.9 with a range of 18 to 110 years. The length of stay in hospital ranged between 1 and 140 days with a mean of 13.5 ± 13.9 days and a median of 10 days. 33 of the subjects were single, 406 were married, 94 were widowed (11 males and 83 females) and 4 were divorced. 46.7% (251) were admitted for CVA and 30.9% (166) for heart failure. Cardiomyopathy/valvular heart diseases (clinical diagnosis due to absence of echocardiography) constituted 3.9%, hypertension 20.5% and pre-existing hypertension with uremia 1.9%.

**Conclusion:**

The study has shown that cardiovascular disease contributed significantly to medical admissions the elderly accounting for a significant proportion. There is thus the need for intensification of primary preventive strategies for cardiovascular diseases.

## Introduction

Cardiovascular disease (CVD) is a broad term used to describe a range of diseases that affect the heart and the circulatory system. Heart disease develops as a result of complex interactions between genes and environment. [1–3] The most frequent forms of CVD are coronary heart disease and stroke, and other forms include hypertensive heart disease, arrhythmia and heart failure [4].

Information on disease prevalence in communities is of vital importance and cardiovascular disease (CVD) is one of the top killer diseases and is problematic for both developed and developing countries.

As a result of the epidemiologic transition, chronic diseases especially cardiovascular diseases including stroke, hypertension and diabetes mellitus are attaining prevalence of heightened proportion [5–7]. This prevalence is attested to by the preponderance of chronic non-communicable diseases in various hospitals across developing countries including Nigeria which has also been documented [8–12].

Cardiovascular disease mortality is top in the rank of mortality of related disease in both developing and developed countries [13, 14]. In developed countries heart disease is still the leading cause of death followed by cancer [15, 16] despite the availability of more sophisticated technologies for the diagnosis and treatment and better management of CVDs, unlike in the developing countries where these are limited. There have been previous reports on pattern of CVD admissions from hospitals in Nigeria, however none has come from Nnamdi Azikiwe University Teaching Hospital (NAUTH), Nnewi and it is with this background that this retrospective study was carried out in Nnamdi Azikiwe University Teaching Hospital Nnewi (NAUTH).

NAUTH is a tertiary hospital serving all the towns of Anambra, parts of Imo, Delta and Enugu States. There are 2 medical wards of 30 beds each, one for males and one for females. Admissions are mostly through the Accident and Emergency (A&E) department and the medical out-patient clinics. Those admitted were aged 18 years and above. The aim of this study therefore is to provide information on the pattern and burden of cardiovascular admissions in a tertiary health care facility in South East Nigeria.

## Methods

A retrospective study of the pattern of cardiovascular admission of patients in the medical wards of the NAUTH, Nnewi over a three year period from January 2007 to December 2009 was conducted. The patients’ case notes were retrieved, bio-data, final diagnosis (as made by the managing specialist) after patients have been investigated, and the final outcome were entered into a pre-coded spreadsheet. Case notes of patients without adequate clinical records were excluded from the study. Analyses were made of the various diagnoses and outcome; length of hospitalization etc. Data was analyzed with the aid of the Statistical Package for Social Sciences (SPSS Inc, Chicago, IL Version 13). Data were expressed as frequency and percentages. Ethical clearance for the study was obtained from the Ethical Committee of the Nnamdi Azikiwe University Teaching Hospital.

## Results

Over the 3 year period a total of 3546 patients were admitted into the medical wards (male 1790 (50.5%) and females 1756 (49.5%) out of which 537{15%} patients were admitted into the study. Of these 322 (60%) were males and 215 (40%) were females giving a male/female ratio of 1.5:1 p < 0.000001. 359(67.5%) were discharged, 170 (32%) (92 males and 78 females p < 0.014) died and 8 (0.5%) were discharged against medical advice of which 2 were patients with cerebrovascular accident (CVA).

Of the 170 that died the majority of the deaths 105(61.8%), consisting of 57 males and 48 females, were in patients with CVA. Most of the deaths (111 or 65.3%) occurred within the first seven days of admission. The mean age of the population was 60.7 years ±15.9 with a minimum of 18 years and maximum of 110 years. There was no statistical difference between the ages of the male and female patients p =0.193.

The length of stay in hospital ranged between 1 and 140 days with a mean of 13.5 ± 13.9 days and a median of 10 days. There was no statistical difference in the duration of stay between the male and female patients p = 0.397. Table 1 shows the marital status of the patients. Table 2 shows the pattern of cardiovascular admissions into medical wards. Table 3 shows the age distribution of the patients with those aged 50 years and above constituting 77.65% of the admissions. Table 4 shows the various cardiovascular causes of admission by sex.

**Table 1:** Marital status of patients

		Sex of patients			
		Male	Female	Total	Per cent (%)
**Marital Status**	**Single**	20	13	33	6.15
	**Married**	290	116	406	75.6
	**Widowed**	11	83	94	17.5
	**Divorced**	1	3	4	0.74
	**Total**	322	215	537	100

**Table 2:** Cardiovascular disease that led to the admission

CVD that brought about admission	Number	Per cent (%)
CVA	251	46.7
Heart Failure	166	30.9
Anemic	11	(2)
Cardiomyopathy/VHD	(21)	(3.9)
Hypertensive	(134)	(25)
Hypertension	110	20.5
Hypertension/Uremia	10	1.9

CVD= cardiovascular disease, CVA = cerebrovascular accident, VHD = vavular heart disease.

**Table 3:** Age distribution of the patients

Age group (years)	Frequency	Percent (%)
<20	4	0.74
20-29	18	3.35
30-39	29	5.4
40-49	69	12.85
50-59	118	21.97
60-69	122	22.72
70-79	108	20.11
>80	69	12.85
**Total**	**537**	**100**

**Table 4:** Cardiovascular causes of admission by sex

	Sex of patient		Total
	Male	Female	
**Reason for admission**			
Anemic Heart Failure	4	7	11
Cardiomyopathy/VHD	13	8	21
Hypertensive Heart Failure	81	53	134
CVA	140	111	251
Hypertension	79	31	110
Hyppertension/Uremia	5	5	10

CVA = cerebrovascular accident, VHD = valvular heart disease.

## Discussion

The pattern of admission in a hospital is of utmost importance in terms of the epidemiology of diseases in that environment as well as in the provision of equipment, drugs, and manpower in such health institutions.

This study has shown that cardiovascular disease is common in persons admitted into the medical wards of NAUTH, Nnewi and that it is commoner in those aged 50 years and above. CVDs consisted of 15% of total admissions over the 3 year period that was studied. In an earlier work in NAUTH on pattern of admissions in the medical wards Osuafor and Ele [17] got 30.3% of total admissions when the per centages for heart failure (12.3%), Hypertension (10.6%) and Cerebrovascular accident (7.4%) are added together which is twice as high as our rate of 15%. The explanation could well be that at the time of the study of Osuafor and Ele (December 1990-December 1992) HIV-AIDS which could have taken up bed-space and thus reduced the percentage of cardiovascular admission, had not become what it is today as there was no mention of it in the table of indication for admission of that study [17]. It is also lower than the 19.4% reported by Ansa et al [12] and Chen et al in Shanghai China [18] but much higher than the 8.2%of Amendezo et al [19]. It agrees with the 16% reported by Reitsma et al [20]. The higher incidence of CVD among patients older than 50 years and above is in keeping with the general trend of NCD of which CVD is one being more common with increasing age [21]. This can be explained by the fact that longevity prolongs the time to exposure to risk factors resulting in a greater probability of CVD in the older age groups as shown by Al Mamun et al [22] as they stated that high risk profile at middle age shortens the duration of life.

More males (60%) were admitted compared to females (40%). This is in agreement with the study of Okunola et al [23], as well as the study of Unachukwu CN et al that had male: female ratio of 1.7:1 [24]. An explanation of the preponderance of males in cardiovascular admissions could well be due to our socio-cultural values in which the society places higher values on males and thus more anxious to take them to hospital when they fall sick as it is considered that his survival is important to the continued existence of the family lineage. It could also be that the males are financially more empowered than the females and therefore could afford the cost of hospital treatment. It is also worth mentioning that symptoms of heart disease are atypical in women [25] and may partly explain the preponderance of males in cardiovascular admissions.

The mean age of the population was 60.7±15.9 and there was no statistically significant difference between the ages of the male and female patients. The mean age in this study is lower than that obtained by Smith and Mensah [26] but higher than that of Ansa [11] which was 52±12.7 and Amendezo et al [19] with a mean age of 47.17±16.04. As in Western countries aging had being used to explain the high CVD prevalence and mortality. [27–29] Lakata and Levy [30] had observed that the incidence and prevalence of these diseases increased steeply with advancing age. Furthermore not only does clinically overt cardiovascular disease increase dramatically with aging, but so do sub clinical or occult diseases, such as silent coronary atherosclerosis.

CVA was the commonest cardiovascular disease warranting admission into the medical wards contributing 46.7% of those admitted and corroborates the finding in other studies [8, 23] where CVA was found to be a major cause of cardiovascular admissions. The large number of patients admitted on account of CVA reflects the poor awareness of the risk factors particularly hypertension and diabetes mellitus. It may also be a reflection of the belief that CVA is an illness that comes because the gods are angry. The high mortality recorded in patients with CVA may be because the patients did not seek attention early enough. Amendezo et al [19] had earlier shown a mean period of 118 days between the occurrence of clinical signs and the start of treatment. In the area of study most people believe that CVA is a result of a spell cast on the patient by supposed enemies or as a result of a curse by the gods thus they first seek help from the traditional medicine practitioners before coming to orthodox hospital. This delay in seeking treatment contributes to the poor prognosis [18]

Hypertension as a cause of CVD admission ranked third. However when taken together with hypertensive heart failure and uremia secondary to hypertension, hypertension becomes the commonest cause of cardiovascular admission at 47.4%. Earlier studies in Nigeria had given rates of 32.3 to 36.9% [31, 32]. Rates of 34.1% and 22.4% were reported in Lusaka Zambia and Tanzania respectively [33, 34].

Hypertension according to the NCD [21] has a prevalence of 10% in Nigeria and they had used the cut-off of systolic ≥160mmHg and of diastolic ≥95mmHg which values are higher than current cut-off points. There are newer reports on the prevalence of hypertension. The studies of Osuji et al [35] and Ahaneku et al [36] had rates of 44.5% which are much higher than NCD rate. The high rate of hypertension admission could be as a result of poor awareness and poor control of hypertension as Osuji et al [35] had shown that only 43.7% of hypertensives were aware of their hypertension and of these only 13% had BP control of ≤ 140/90mmHg, while Lindblad et al [37] in the Netherlands state that 33% were unaware of their hypertension 36% were aware and uncontrolled and 31% were aware and controlled. Working in the USA, Whelton et al [38] stated that only 55% of USA hypertensives who were aware of their diagnosis were on treatment and only 29% were controlled. Uncontrolled hypertension is associated with several complications such as heart failure, ischemic heart disease, stroke, chronic renal failure and others [39]. Furthermore hypertension often co-exists with other potent cardiovascular risk factors, thus increasing the risk of early death from cardiovascular causes by about three fold. [40] Men are known to die at an earlier age than women. [41, 42]

Heart failure accounted for 30.9% of all the cardiovascular admissions in this study. Heart failure is said to account for 3-7% of all admissions in Africa [43] and is fast becoming the global disease as the prevalence is increasing at an alarming rate worldwide [44]. Majority were as a result of hypertension followed by anemia and cardiomyopathy/ valvular heart disease. It should be noted that cardiomyopathy/valvular heart disease were grouped together because at the time of the study echocardiography machines were not available in the hospital and it was not possible to clearly distinguish between the 2 in all cases. The large number of patients admitted for heart failure may be a reflection of poor awareness of risk factors and early symptoms of heart failure. It may also be attributed to late treatment or lack of access to early cardiovascular care.

The marital status of the subjects showed that 33 were single, 406 were married, 4 were divorced and 94 were widowed. Of the widowed 83 were females and 11 males. This high ratio of widowed women over widowed men reflects the culture of women not remarrying after the death of their husbands especially if they had children for their late husbands or were past child bearing age. For the men the society expects them to remarry in order to get somebody to help look after the children from the late wife and or to have more children.

## Conclusion

The study has shown that cardiovascular disease contributed significantly to medical admissions over the 3 year period of the study with the elderly accounting for a significant proportion. There is thus the need for intensification of primary preventive strategies for cardiovascular diseases. There is also the need to strengthen the capacity of secondary health to accommodate less severe CVD cases. The set-up of health education mechanisms to create awareness and early adequate management can reduce the incidence of complications and lower mortality associated with CVDs.

## References

[1] Romanoski CE, Lee S, Kim MJ, Ingram-Drake L, Plaisier CL, Yordanovu R (2010). Systems Genetics Analysis of Gene-by-Environment Interactions in Human Cells. Am J Hum Genet..

[2] Talmud PJ (2007). Gene-Environment interaction and its impact on coronary heart disease risk. Nutr Metab Cardiovasc Dis.

[3] Sing CF, Stengard JH, Kardia SL (2003). Genes, Environment, and Cardiovascular Disease. Arterioscler Thromb Vasc Biol..

[4] WHO Media Centre (2011). Cardiovascular Diseases (CVDs), Fact Sheet No 317.

[5] Omran AR (2005). The Epidemiological Transition: A theory of the of Epidemiology Population change (1971). Milbank Q.

[6] WHO (2002). The World Health Report 2002 Reducing risks promoting healthy life.

[7] Whelton PK, Brancati FL, Appel LJ, Klag MJ (1995). The challenge of hypertension and atherosclerotic cardiovascular disease in economically developing countries. High Blood Press.

[8] Ogun SA, Adelowo OO, Familoni OB, Jaiyesimi AE, Fakoya EA (2000). Pattern and outcome of medical admission at the Ogun State University Teaching Hospital, Sagamu- a three year review. West Afr J Med.

[9] Odenigbo CU, Ogujiofor OC (2009). Pattern of medical admission at the Federal Medical Centre, Asaba: A Two Year Review. Nig J Clin Pract.

[10] Ike SO (2008). The Pattern of admission into the Medical Wards of the University of Nigeria Teaching Hospital, Enugu. Nig J Clin Pract.

[11] Ansa VO, Ekott JU, Bassey EO (2008). Profile and outcome of cardiovascular admissions at the University of Uyo Teaching Hospital, Uyo: a five year review. Niger J Clin Pract..

[12] Sanya EO, Akande TM, Opadijo G, Olarinoye JK, Bojuwoye BJ (2008). Pattern and outcome of medical admission of elderly patients seen at University of Ilorin Teaching Hospital, Ilorin. Afr J Med Med Sci..

[13] Foot DK, Lewis RP, Pearson TA, Beller GA (2000). Demographics and Cardiology, 1950-2050. J AM Coll Cardiol..

[14] National Centre for Health Statistics, United States (1996). 1995 DHHS Pub no (PHS) 96-1232.

[15] Petersen S, Peto V, Rayner M, Leal J, Luengo-Fernandez R, Gray A (2005). European Cardiovascular Disease Statistics.

[16] Mathers CD, Boerma T, Fat DM (2009). Global and Regional Causes of Death. Br Med Bull..

[17] Osuafor TO, Ele PU (2004). The Pattern of admissions in the Medical Wards of Nnamdi Azikiwe University Teaching Hospital (NAUTH) Nnewi. Orient Journal of Medicine.

[18] Chen HZ, Fan WH, Jin XJ, Wang Q, Zhou J, Shi ZY (2003). Changing trends of etiologic characteristics of cardiovascular diseases among inpatients in Shanghai: A retrospective observational study from 1948 to 1999. Zhonghua Nei Ke Za Zhi..

[19] Amendezo E, Twagirumukiza M, Sebantunzi O, Kagame A (2008). Inhospital Cardiovascular morbidity and mortality in the department of internal Medicine at CHU Kigali (Rwanda). Ann Trop Med Public Health..

[20] Reitsma JB, Dalstra JA, Bonsel GJ, van de Meulen JH, Koster RW, Gunning-Schepers LJ (1999). Cardiovascular disease in the Netherlands, 1975 to 1995:Decline in mortality, but increasing numbers of patients with chronic conditions. Heart.

[21] Akinkugbe OO (1997). (ED) Non-communicable disease in Nigeria: final report of a national survey.

[22] Abdullah Al Mamun A, Peeters A, Willekens F, Bonneux L Impact of Cardiovascular Disease Risk Factors in Middle Age on Later Ages of Life: A Life Course.

[23] Okunola OO, Akintunde AA, Akinwusi PO (2011). Some emerging issues in medical admission pattern in the tropics. J Dent Med Med Sci..

[24] Unachukwu CN, Agomuoh DI, Alasia DD (2008). Pattern of non- communicable diseases among medical admissions in Port Harcourt, Nigeria. Niger J Clin Pract..

[25] Mosca L, Manson JE, Sutherland SE, Langer RD, Maniolo T, Barret-Connor E (1997). Cardiovascular Disease in Women: A Statement for Healthcare Professionals from The American Heart Association. Circulation.

[26] Smith SM, Mensah GA (2003). Population aging and implications for epidemic cardiovascular disease in Sub-Saharan Africa. Ethn Dis.

[27] Mackay J, Mensah GA (2004). Atlas of heart disease and stroke.

[28] Mensah GA, Brown DW (2007). An overview of cardiovascular disease burden in the United States. Health Aff (Millwood).

[29] SoRelle R (1999). Global epidemic of cardiovascular disease expected by the year 2050. Circulation.

[30] Lakatta EG, Levy D (2003). Arterial and Cardiac Aging: Major Shareholders in Cardiovascular Disease Enterprises: Part I: Aging Arteries: A “Set Up” for Vascular Disease. Circulation.

[31] Brockington IF, Edington GM (1972). Adult heart disease in Western Nigeria: A clinicopathological synopsis. Am Heart J.

[32] Ladipo GO, Froude JR, Parry EH (1977). Pattern of heart disease in adults of the Nigerian savanna: A prospective clinical study. Afr J Med Med Sci..

[33] Obineche EN (1976). Pattern of cardiovascular disease in Lusaka - A review. East Afr Med J.

[34] Vaughan JP (1977). Cardiovascular diseases seen in Tanzanian hospitals 1966 to 1968. East Afr Med J.

[35] Osuji CU, Nzerem BA, Meledu SC, Dioka CE, Nwobodo E, Amilo G (2006). Hypertension prevalence and awareness amongst a group of women attending “August Meeting”. Journal of Biomedical Research.

[36] Ahaneku GI, Osuji CU, Anusiuba BC, Ikeh VO, Oguejiofor OC, Ahaneku JE (2011). Evaluation of blood pressure and indices of obesity in a typical rural Community in Eastern Nigeria. Ann Afr Med..

[37] Lindblad U, Ek J, Eckner J, Larsson CA, Shan G, Rastam L (2012). Prevalence awareness, treatment, and control of hypertension: Rule of in the Skaraborg project. Scand J Prim Health Care.

[38] Whelton PK, He J, Muntner P (2004). Prevalence, awareness, treatment and control of hypertension in North America, North Africa and Asia. J Hum Hypertens.

[39] Kadiri S, Walker O, Salako BI, Akinkugbe O (1999). Blood pressure, hypertension and clinical correlates in urban workers in Ibadan, a revisit. J Hum Hypertens.

[40] Dirks J, Robinson S (2006). Preventing vascular diseases in the emerging world: a multidisciplinary approach. Diabetes Voice.

[41] Springer KW, Mouzon DM (2011). “Macho Men” and preventive health care: implications for older men in different social classes. J Health Soc Behav..

[42] Pinkhasov RM, Shteynshlynger A, Hakimian P, Lindsay GK, Samaki DB, Shabsigh R (2010). Are men shortchanged on health? Perspective on life expectancy, morbidity, and in men and women in the United States. Int J Clin Pract..

[43] Oyoo GO, Ogola EN (1999). Clinical and socio-demographic aspects of congestive heart failure patients at Kenyatta National Hospital. Nairobi. East Afr Med J.

[44] Sanderson JE, Tse TF (2003). Heart Failure: a global disease requiring a global response. Heart.

